# ImputEHR: A Visualization Tool of Imputation for the Prediction of Biomedical Data

**DOI:** 10.3389/fgene.2021.691274

**Published:** 2021-07-02

**Authors:** Yi-Hui Zhou, Ehsan Saghapour

**Affiliations:** ^1^Department of Biological Science, North Carolina State University, Raleigh, NC, United States; ^2^Bioinformatics Research Center, North Carolina State University, Raleigh, NC, United States

**Keywords:** electronic health records, imputation, gradient boosting, prediction, decision trees

## Abstract

Electronic health records (EHRs) have been widely adopted in recent years, but often include a high proportion of missing data, which can create difficulties in implementing machine learning and other tools of personalized medicine. Completed datasets are preferred for a number of analysis methods, and successful imputation of missing EHR data can improve interpretation and increase our power to predict health outcomes. However, use of the most popular imputation methods mainly require scripting skills, and are implemented using various packages and syntax. Thus, the implementation of a full suite of methods is generally out of reach to all except experienced data scientists. Moreover, imputation is often considered as a separate exercise from exploratory data analysis, but should be considered as art of the data exploration process. We have created a new graphical tool, ImputEHR, that is based on a Python base and allows implementation of a range of simple and sophisticated (e.g., gradient-boosted tree-based and neural network) data imputation approaches. In addition to imputation, the tool enables data exploration for informed decision-making, as well as implementing machine learning prediction tools for response data selected by the user. Although the approach works for any missing data problem, the tool is primarily motivated by problems encountered for EHR and other biomedical data. We illustrate the tool using multiple real datasets, providing performance measures of imputation and downstream predictive analysis.

## 1. Introduction

Recently, hospitals in the United States have made a concerted effort to transition their health records from paper to digital, the proportion of which has dramatically increased, from 9.4% in 2008 to 75.5% in 2014 (Charles et al., 2013). Although we are seeing improvements in the overall quality of EHR-derived datasets, data missingness remains a substantial and unavoidable issue (Chan et al., 2010; Weiskopf and Weng, 2013). Missing EHR data could be caused by a lack of collection or a lack of documentation (Wells et al., 2013), and it could be missing at random or not at random (Hu et al., 2017). Researchers have noted the problems posed by missing data and are developing strategies to address it (Haukoos and Newgard, 2007; Newgard and Haukoos, 2007), as EHR systems become more relevant and adopted worldwide.

The expectation of collecting real-world data without missingness is unrealistic. Even the most detailed protocols for data collection cannot guarantee that every subject will have a record at each observation. Missing data present a challenge for analysts, as it can introduce a substantial amount of bias, makes the handling and analysis of the data more arduous, and creates reductions in efficiency (Barnard and Meng, 1999). Many standard analysis methods, including regression, are defeated by even a single missing value from among many potential predictors. Thus, it is possible that standard analysis may essentially “throw away” large portions of the data, even though a small fraction of the data may actually be missing. Ultimately, data missingness decreases our ability to discern the deeper structures and relationships underlying the observations, causing a significant negative impact on scientific research (McKnight et al., 2007). Many important scientific and business decisions are based on results from data analyses, and so dealing with missing data in an appropriate manner is recognized as a crucial step.

The process of data *imputation* (artificially replacing missing data with an estimated value) offers a practical work-around so that many downstream data handling steps become feasible. This process preserves all observations by replacing missing data with an estimated value based on other available information. Once all missing values have been imputed, datasets can then be analyzed using standard techniques for complete data (Gelman and Hill, 2006). Many advanced analysis methods, such as machine learning, require a complete dataset, so imputing missing data enables researchers to apply statistical and computational association methods that would otherwise be unavailable. Missing data imputation methods are considered standard in areas such as genetic association (Schurz et al., 2019) and proteomics (Jin et al., 2021), where correlation structures are strong. For electronic health records, the need for imputation methods have more recently realized (Jazayeri et al., 2020), and the use of imputation shown to improve prediction accuracy (Beaulieu-Jones et al., 2017). However, use of many of these methods requires purpose-built scripting pipelines (Hu et al., 2017), while we aim in this paper to provide a variety of tools using a very simple interface.

When imputation is performed, issues of bias and correct handling of variability/uncertainty arise (Rubin, 2003), depending on the imputation accuracy. Much of the traditional statistical literature on handling missing data has dealt with likelihood inference for low-dimensional problems (Rubin, 1976), or resampling techniques such as multiple imputation, which can mimic and account for imputation uncertainty. However, our focus here is on the practical impact of imputation for downstream analysis, such as EHR-based prediction of important health measures. For such efforts, the emphasis is placed on the success of machine-learning methods, which themselves may involve penalization techniques and estimation known to be biased. Thus, we consider imputation as a possibly essential pre-processing step to serve a larger goal, and it should be judged accordingly. Machine-learning methods have reached a high degree of sophistication in biology and genomics (Le and Huynh, 2019; Le et al., 2019), but for electronic health records, which tend to be less structured, a variety of approaches must be considered. In this work, we evaluate the effectiveness of various imputation methods on EHR and other real-world datasets, and proposed a practical and fast imputation method as a hybrid of existing methods.

## 2. Datasets

### 2.1. MIMIC-III

The Medical Information Mart for Intensive Care III (MIMIC-III) is a large database comprising de-identified health-related data associated with over 40,000 patients who stayed in ICUs at the Beth Israel Deaconess Medical Center between 2001 and 2012 (Johnson et al., 2016). MIMIC-III is freely available on PhysioNet (https://mimic.physionet.org). The database includes information such as demographics, hourly vital sign measurements, laboratory test results, procedures, medications, caregiver notes, imaging reports, and mortality (including post-hospital discharge).

MIMIC-III is disseminated as a relational database consisting of 26 tables containing many categorical and continuous features. We extracted ICD-9 codes from the “DIAGNOSES_ICD” table, demographics and discharge time or time of death from the “ADMISSIONS” table, and laboratory measurements from the “LABEVENTS” table with <30% missing, totaling 603 features. ICD-9 is the actual code corresponding to the diagnosis assigned to the patient. However, it is often unclear whether a negative value indicates that the patient does not have a specific code, or the code is truly missing. The laboratory measurements are continuous values for 726 unique items. The missing proportion of laboratory tests can be as high as 90%, which significantly impacts any downstream analysis of these data. Therefore, it is important to study the appropriate missing data imputation methods for laboratory tests.

### 2.2. Datasets From the UCI Machine Learning Repository

The UCI Machine Learning Repository is a collection of datasets that are used by researchers for the empirical analysis of machine learning algorithms (Dua and Graff, 2017). Although these datasets are largely complete, we can effectively evaluate our imputation under complete missing at random assumptions by artificially masking individual observations and recording the imputation accuracy. Datasets are maintained on their website (https://archive.ics.uci.edu/ml/index.php). We selected the following four datasets for imputation testing: (1) “Boston,” information for predicting the value of house prices (Harrison and Rubinfeld, 1978); (2) “Spam,” attributes to determine whether e-mails were spam (Cranor and LaMacchia, 1998), (3) “Letter,” character image features to identify a letter of the alphabet (Frey and Slate, 1991), and (4) “Breast Cancer,” numerical features of cell images for tumor diagnosis in 357 malignant and 212 benign samples (Street et al., 1993). These datasets have varying numbers of samples and features, with both continuous and categorical data, as summarized in Table 1.

**Table 1:** The Boston data have information for predicting the value of house prices; the spam data contain the attributes to determine whether e-mails spam; the letter data have character image features to identify a letter of the alphabet; the breast cancer data gathered the numerical features of cell images for tumor diagnosis.

**Dataset**	**Download link**	**# Sample**	**# Features**	**Attribute type**
Boston	https://archive.ics.uci.edu/ml/machine-learning-databases/housing	506	13	Both
Spam	https://archive.ics.uci.edu/ml/datasets/Spambase	4,601	57	Continuous
Letter	https://archive.ics.uci.edu/ml/datasets/Letter+Recognition	20,000	16	Categorical
Breast cancer	https://archieve.ics.uci.edu/ml/datasets/Breast+Cancer+Wisconsin+%28Diagnostic%29	569	30	Continuous

## 3. Methods

ImputEHR is designed to provide several existing imputation methods in easy-to-use interface, as described below. In addition, we have noted that tree-based imputation has been relatively under-represented, and we propose some novel enhancements here in order to provide effective tree-based imputations with reasonable computational burden. Gradient boosted trees are an effective machine learning algorithm that iteratively combines decision trees in order to make predictions. In Python, we modified the MissForest algorithm (Stekhoven and Bühlmann, 2012), which imputes missing values using random forests (Liaw and Wiener, 2002), by applying the *LightGBM* module, a gradient boosting framework known for its light computational burden and better performance than previous decision tree-based algorithms (Ke et al., 2017), in the *missingpy* Python library for missing data imputation. Pseudocode for the ImputeEHR1 algorithm is shown in Table 2. The ImputeEHR2 approach is using the *XGBoost* (Extreme Gradient Boosting) module (Chen et al., 2015), a common boosting algorithm, in the *missingpy* library. The performance of ImputeEHR was validated using MIMIC-III and the four repository datasets.

**Table 2:** Pseudocode of the ImputeEHR algorithm.

**Algorithm: ImputeEHR algorithm**
Require: X is *n* × *m*-dimensional data matrix, with stopping criterion γ
1. Make initial guess using mean or median imputation for missing values;
2. *k* ← *A* sorted indices vector according to t he amount of missing values of
column *X*;
w.r.t. increasing amount of missing values;
3. **While** not γ **do**
4. $X_{old}^{imp}\leftarrow$ Store previously imputed matrix;
5. **for** s in k **do**
6. Fit a LightGBM or Xgboost : $y_{obs}^{\left(s\right)}\sim X_{obs}^{\left(s\right)}$;
7. Predict $y_{miss}^{\left(s\right)}$ using $X_{miss}^{\left(s\right)}$;
8. $X_{new}^{imp}\leftarrow$ update imputed matrix from $y_{miss}^{\left(s\right)}$;
9. **end for**
10. Update γ
11. **end** while
12. **Return** Matrix *X*;

### 3.1. Imputing Missing Data

We compared our proposed ImputeEHR1, ImputeEHR2, and five state-of-the-art imputation methods in Python: MissForest, MICE (Buuren and Groothuis-Oudshoorn, 2010), KNNImputer (Troyanskaya et al., 2001), SoftImpute (Mazumder et al., 2010), and GAIN (Yoon et al., 2018). In addition, we also performed simple feature-mean and feature-median replacement as the most basic and simple imputation method. KNNImputer is based on k-nearest neighbors algorithm. GAIN adapts the generative adversarial nets framework. The MICE and SoftImpute methods are implemented in the *fancyimpute* Python library. SoftImpute uses an iterative soft-thresholded SVD algorithm and MICE uses chained equations to impute missing values. We used default parameter settings for each method, and parameters for the two ImputeEHR methods are listed in Supplementary Table 1.

In each dataset, we generated missing data (missing completely at random), with rates from 10 to 90% in increments of 10% by randomly removing data and ran the imputation methods. The Root Mean Squared Error (RMSE) was then calculated at each missingness rate in comparison of the values between the real and imputed data. We ran 10 iterations in order to obtain average RMSEs.

Supplementary Tables 2–5 show the average RMSEs for each dataset, with the lowest RMSE at each missingness rate highlighted. Overall, our proposed method significantly outperforms all of the state-of-the-art models. ImputeEHR has the lowest RMSE in 24 out of a possible 36 comparisons, followed by MICE and MissForest methods having 6 and 3, respectively.

### 3.2. Testing Runtimes Between Methods

**Figure 1:**
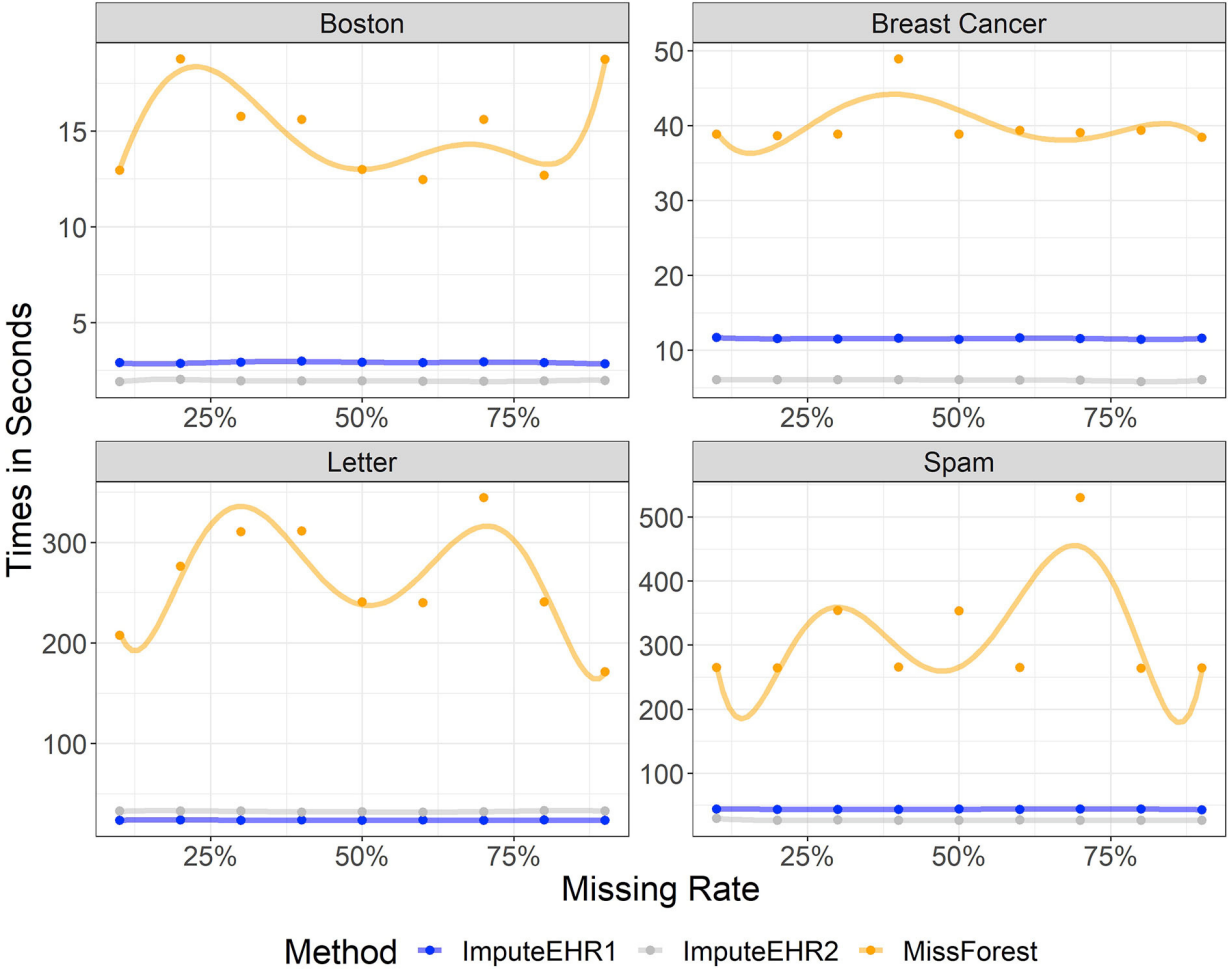
Running time of ImputeEHR1 (blue), MissForest (orange), and ImputeEHR2 (gray) for each dataset.

We evaluated the speeds of ImputeEHR1, ImputeEHR2, and MissForest method, since they are each tree-based learning algorithms, using the *scikit-learn* Python library (Pedregosa et al., 2011). We set the number of trees at 100, and used default values for the remaining parameter settings. Figure 1 shows the runtimes by missingness rate in each dataset. Our experiments show that both ImputeEHR1 and ImputeEHR2 can accelerate the imputation process 20–25 times faster than MissForest while achieving lower RMSEs. Moreover, ImputeEHR1 is faster than ImputeEHR2 for the largest dataset. We performed this experiment on a desktop computer with Windows 10, Intel(R) Xenon CPU E5-2687W v4@3.00 GHz CPU, 128 GB RAM and GeForce GTX 1080, 8 GB.

### 3.3. Evaluating Predictive Performance for a Variable of Interest, After Imputation

**Figure 2:**
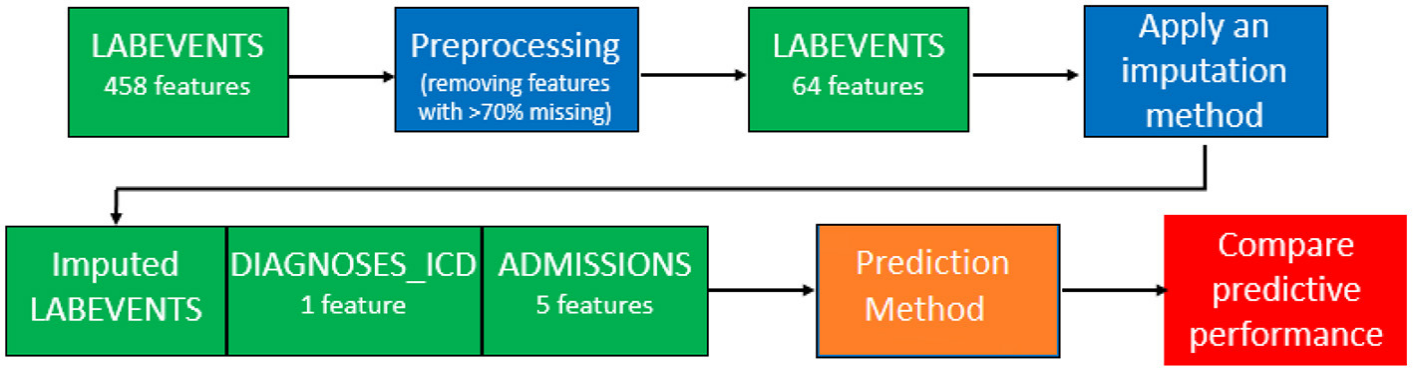
Our pipeline of the MIMIC-III data imputation and prediction.

We attempted to predict the mortality for ICU patients in the MIMIC-III database. Figure 2 provides an illustration of our pipeline. First, we aggregated the laboratory tests in the “LABEVENTS” table by averaging the values taken within the first 24 h of a patient's first admission to ICU. After removing laboratory tests which are >70% missing, 64 items remained. Then, we selected patients with complete records for the 64 laboratory tests, resulting in 714 patients. So our filtered “LABEVENTS” data have dimension 714 patients × 64 laboratory tests, which we used as input for each imputation method.

**Figure 3:**
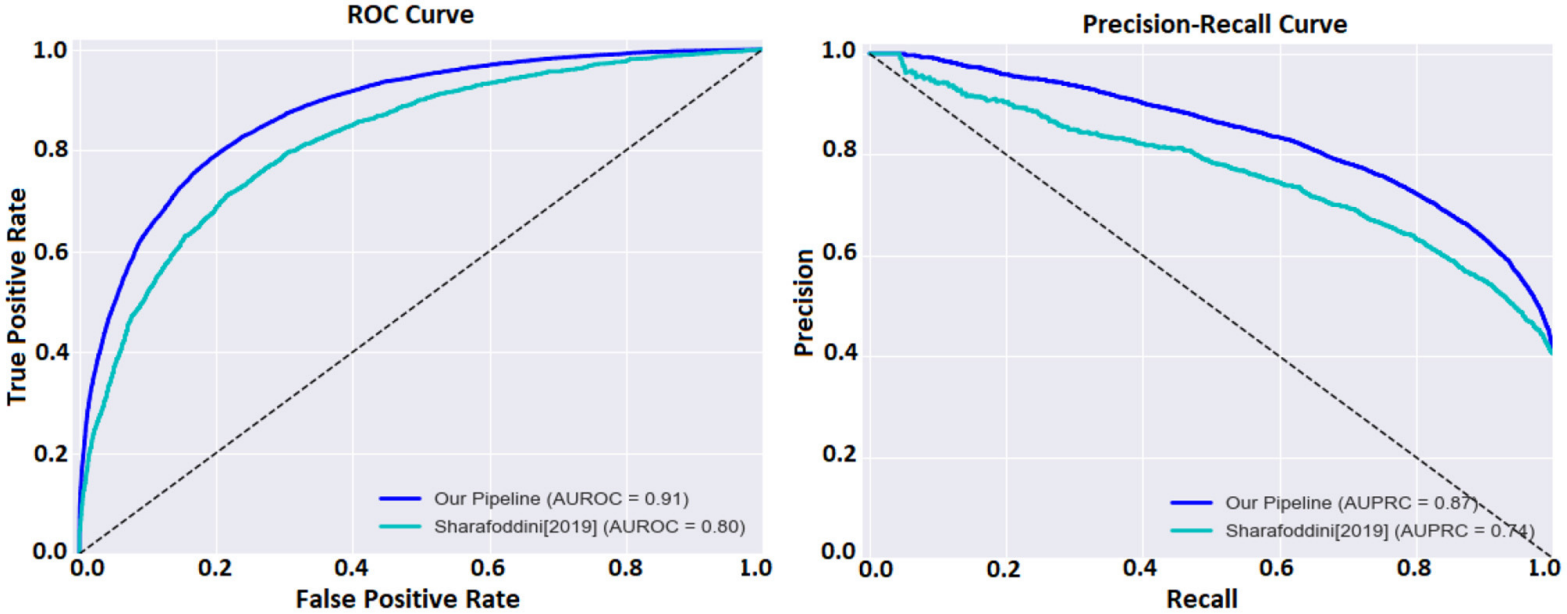
**(Left)** Receiver operating characteristic curve (ROC) comparison between our pipeline and the method (Sharafoddini et al., 2019) on the mortality prediction of the MIMIC-III data. **(Right)** Precision recall curve comparison.

Then, we combined the imputed “LABEVENTS” data with the ICD-9 codes from the “DIAGNOSIS_ICD” table and the demographics and mortality outcome from the “ADMISSIONS” table into a model matrix and applied lasso regression (Tibshirani, 1996) with five-fold cross-validation. This process involves randomly splitting the samples into five groups, keeping four groups as a training set, so the model can predict the outcomes for samples in the fifth group. This process was run five times so outcomes are predicted in all samples. The area under the curve (AUC) is the metric we used to compare the predicted vs. the actual outcomes. The ImputeEHR method has the highest AUC 0.91, and the tree-based algorithms perform better than other methods. Our pipeline provides the highest prediction accuracy comparing the historical mortality prediction in the literature (Sharafoddini et al., 2019), which reached the best AUC 0.80 (Figure 3). Both receiver operating characteristic curve and precision recall curve show that our pipeline provides the best prediction of mortality.

## 4. Web Application

**Figure 4:**
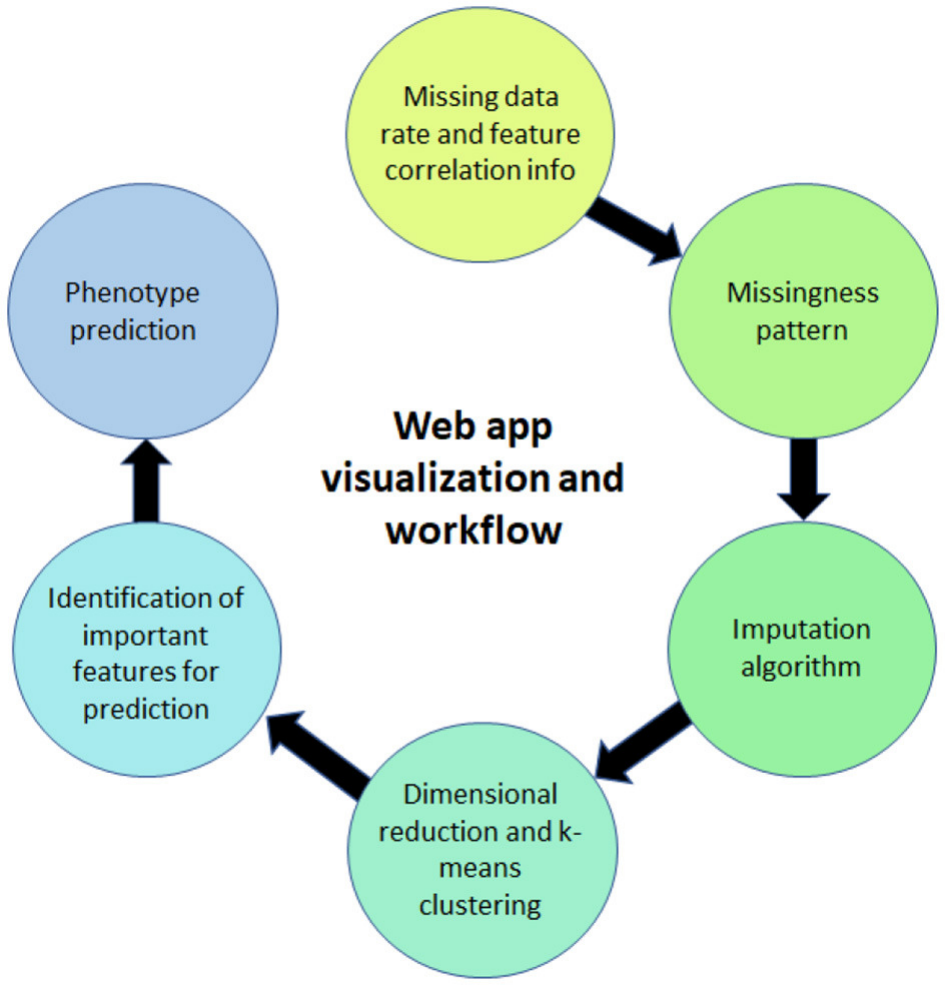
Illustration of the web app for visualization.

The web application (ImputEHR app), available as a scikit-learn package in Python, allows users to apply our pre-processing, feature engineering, and prediction methods on their dataset, and to visualize the results. Below we briefly describe the six major components of the web app, illustrated in Figure 4, and show its capabilities by presenting results of our implementation, using the “Breast Cancer” dataset from the UC Irvine Machine Learning Repository as an example.

### 4.1. Percentage of Missing Rate and Correlation Features Information

Users can obtain initial information about the missing rates of each feature in their dataset. Supplementary Figure 1 shows the percentage of missing values in our example. Since the breast cancer dataset in Table 1 (Street et al., 1993) does not have missing values, we randomly set 35–45% of the values as missing and continue to use it as the toy example for our ImputEHR app.

In addition, the app has the option for users to plot the correlation between any two features (factors). It also helps the users to decide if they need to include these factors that might be highly correlated with each. If the dataset has missing values, users can show the scatterplot before imputing, removing the missing values. Three parameters to better visualize the scatterplot are the color, size, and clarity of the data points (Supplementary Figure 2).

### 4.2. Visualization of Missingness Patterns

As an optional feature in our app, the missingness patterns can be checked by users via the black/white image plot, in which black is for missing values. The user can also hover mouse around the Dendrogram and zoom in to check the information for the grouped factors due to the missingness. Supplementary Figure 3 includes the visualization of Dendrogram on missingness pattern based on the toy data.

### 4.3. Imputation Algorithm

Within the app, the nine imputation methods listed in section 3.1 are available: ImputeEHR1, ImputeEHR2, MissForest, MICE, KNNImputer, SoftImpute, GAIN, mean, and median. Supplementary Table 6 provides the important parameters' selection for the toy example via ImputeEHR1 and ImputeEHR2 methods.

Some methods have their own hyperparameters. For KNNImputer, we set *k* = 5, which is considered the default number of nearest neighbors. Four parameters, “batch_size,” “hint_rate,” “alpha,” and “iteration,” are embedded for the GAIN method. The “batch_size” defines the number of training samples present in a single batch. The “hint_rate” reveals the discriminator partial information about the missingness of the original sample. The “alpha” is a hyperparameter, and “iteration” describes the number of times a batch of data passes through the algorithm to update its parameters.

### 4.4. Visualization From Combining Dimensional Reduction Algorithms and K-Means Clustering

**Figure 5:**
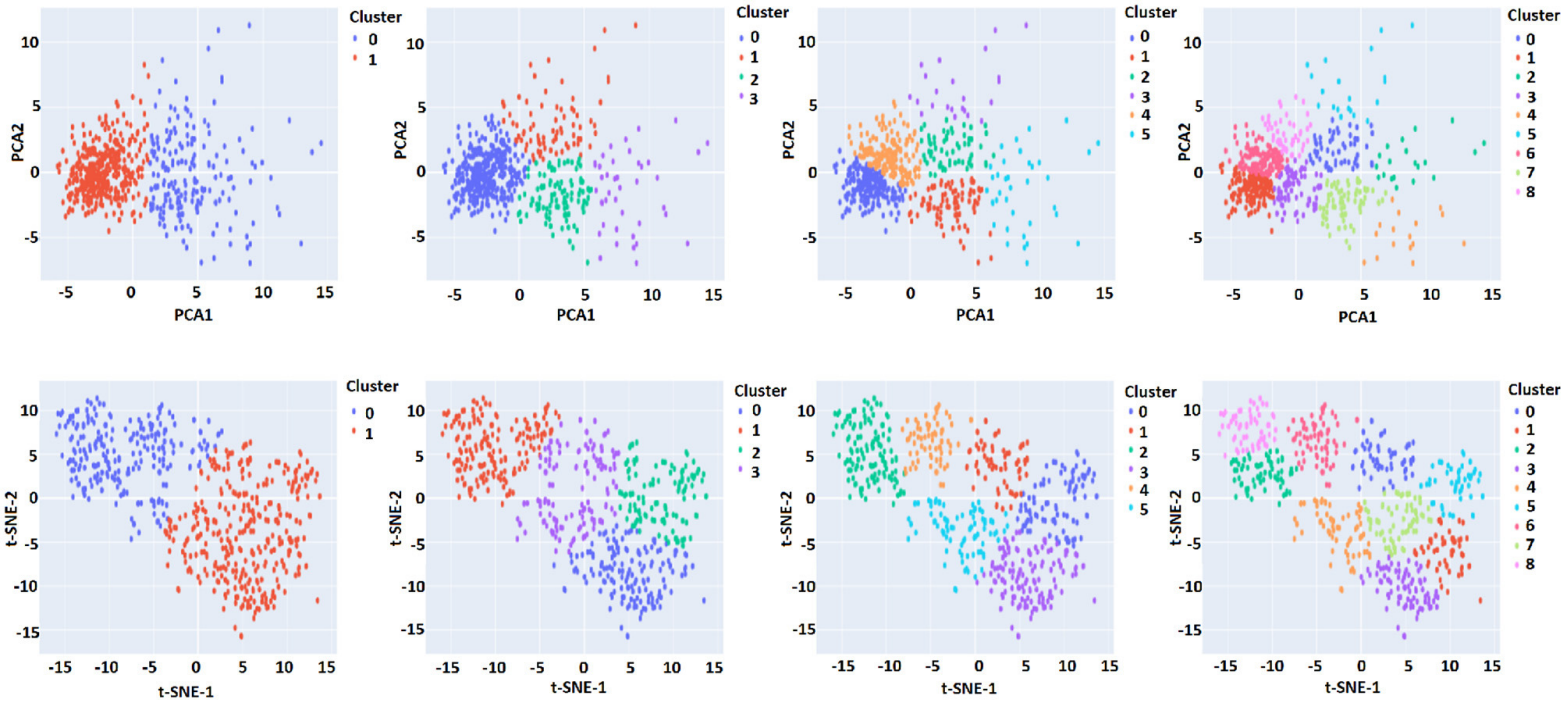
Visualization of patterns in the imputed dataset. User has the option to use the number of cluster and dimension reduction method.

ImputEHR makes it easy for users to visualize patterns in their imputed dataset. Principal component analysis (PCA) Pearson (1901) and t-distributed stochastic neighbor embedding (t-SNE) (Van der Maaten and Hinton, 2008) methods are embedded for dimension reduction. Users can plot the result of either method, partitioning the observations into k clusters. Our ImputEHR app suggests the number of optimal clusters using the Elbow method (Syakur et al., 2018), which runs k-means clustering on the imputed dataset for a range of values for *k* between 1 and 9. For the visualization purpose, the green line in Supplementary Figure 4 indicates the best choice of *k* plot on the toy example. Three parameters considered for the t-SNE method are “learning rate,” “n_iter” (number of iterations), and “perplexity.” Perplexity defines the number of close neighbors at each point, and learning rate affects the convergence of the embedding. In Figure 5 and Supplementary Figure 5, we applied k-means method with different numbers of clusters on the outcome of the PCA and t-SNE methods. In our app, user can also mouse over the point and see which variable it is.

### 4.5. Visualization of the Important Features

**Figure 6:**
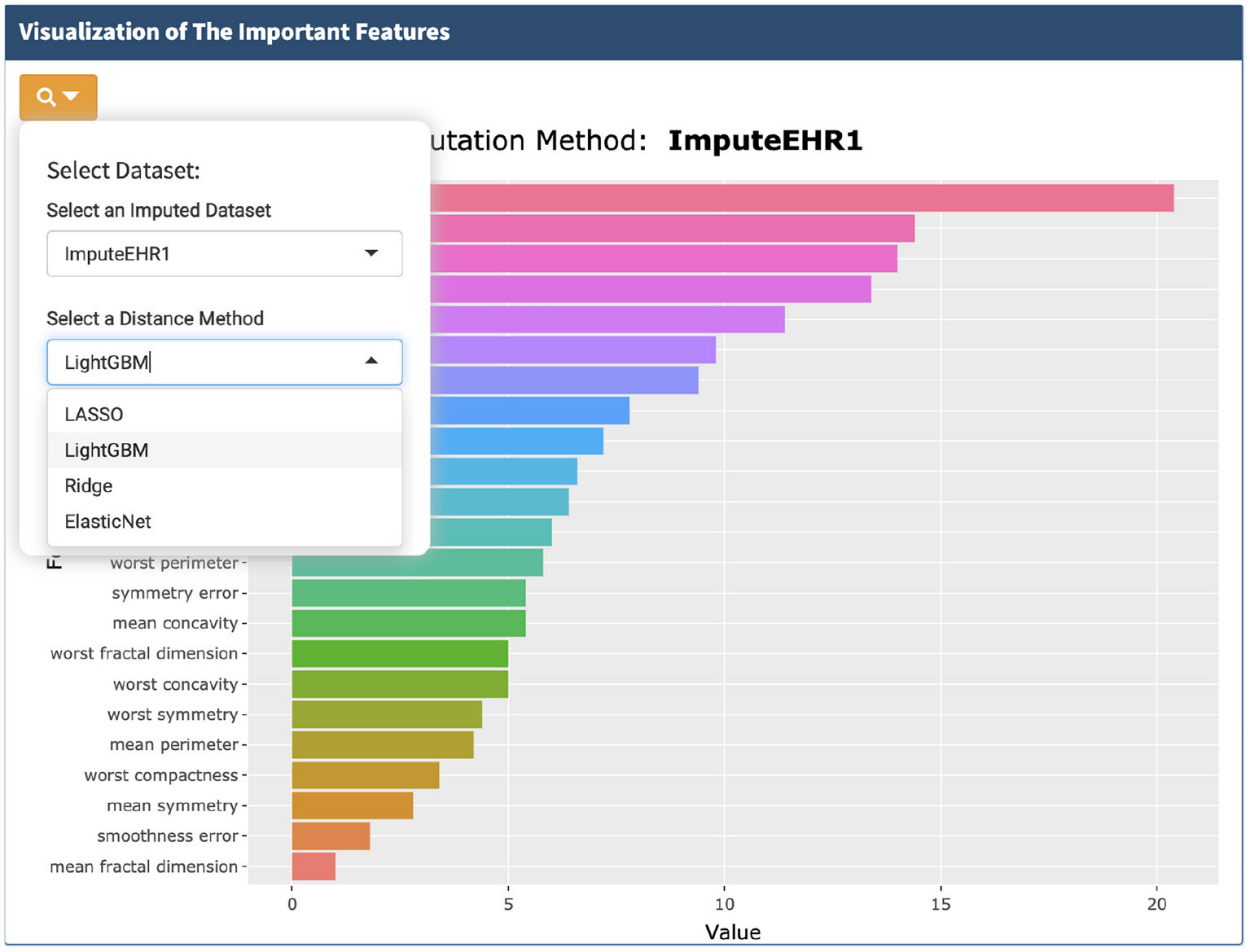
Visualization of the important features selected by the four methods.

A very useful feature of our app is that it helps users to nail down the most important features for further investigation. We provide the users four methods for feature selection from the imputed dataset: LightGBM (Ke et al., 2017), lasso (Tibshirani, 1996), ridge (Hoerl and Kennard, 1970), and elastic net (Zou and Hastie, 2005) (Figure 6). Users can decide how many important features to visualize.

### 4.6. Visualization of the Phenotype Prediction

When performing imputation, if downstream prediction is intended, then the response variable should be removed from the imputation process to avoid overtraining datasets in which cross-validation for prediction of the response must be used. Accordingly, ImputEHR enables the user to select a response variable to be excluded from the imputation process. We also provide the author the visualization of the correlation between the imputed value and the masked 5% non-missing data for each variable (Supplementary Figure 4).

**Figure 7:**
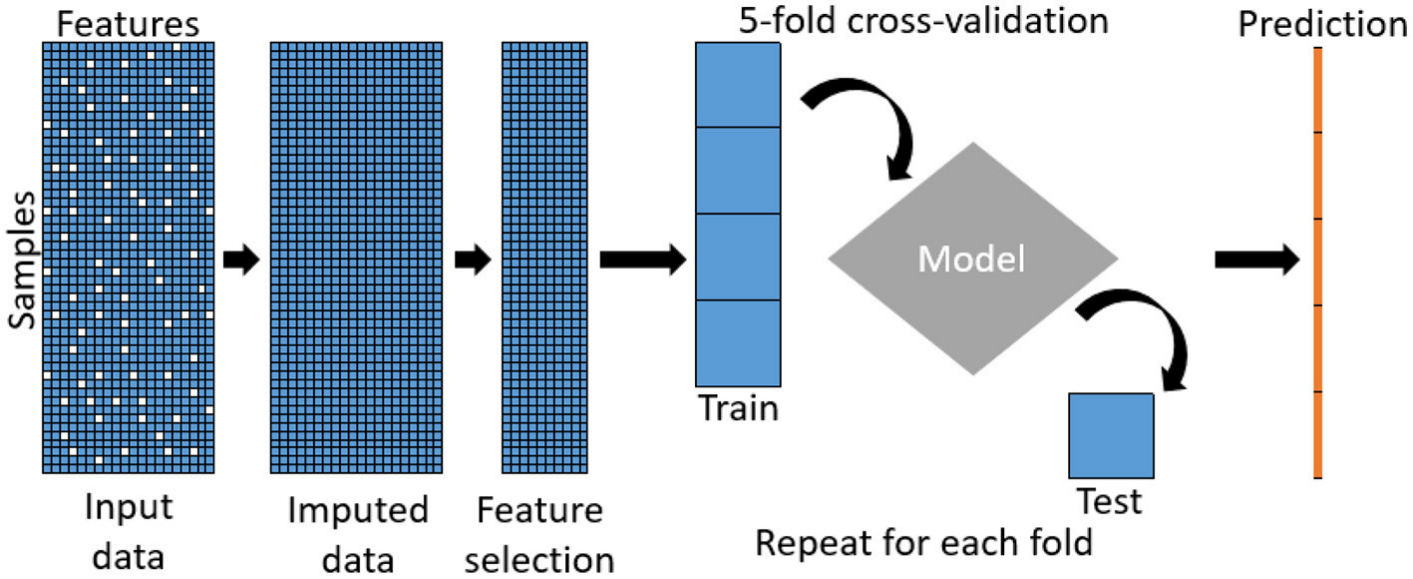
Pipeline of the predictive model.

Important features from an imputed dataset are selected as input to predict the phenotype, illustrated in Figure 7, using five-fold cross-validation to avoid overfitting. Users can select from a suite of prediction methods including random forests, lasso, LightGBM, and KNN.

The running time for a job depends largely on the size of dataset, the missing rate, and the computer hardware. All analyses were performed in Python 3.6.

## 5. Conclusions

ImputeEHR can quickly and accurately impute missing data, implementing a variety of methods. The ease of performing imputation can lead to better predictive performance, as many methods are made feasible by imputation. We have created a tool covering a range of imputation options, including novel and fast tree-based methods. We have also included a variety of basic phenotype prediction methods, although the user can easily output the imputed dataset for import into other prediction routines. As with any imputation tools, the accuracy will be limited by the correlation structures, and in general the number of features relative to the sample size. For these and other reasons, this tool is not designed for genomic imputation (Schurz et al., 2019) or for proteomics data (Jin et al., 2021), or other areas with well-understood biological correlation structures. However, the ease of use and seamless interface for using multiple imputation methods makes our approach a useful approach in a variety of analysis pipelines.

## Data Availability Statement

The original contributions presented in the study are included in the article/Supplementary Material. The toydata for the ImputEHR app is located at https://github.com/zhouLabNCSU/ImputEHR/tree/main/Demo%20File, further inquiries can be directed to the corresponding author/s.

## Author Contributions

Y-HZ is the leader of this project. Her contribution includes writing the manuscript, designing the data analysis, summarizing the results, and software management. ES contributed to the Python code underneath the ImputEHR app. Both authors contributed to the article and approved the submitted version.

## Conflict of Interest

The authors declare that the research was conducted in the absence of any commercial or financial relationships that could be construed as a potential conflict of interest.

## Supplementary Material

The Rshiny link, supplementary documents, and breast cancer toy example dataset are available at: https://github.com/zhouLabNCSU/ImputEHR.
