# Supplementary material for: ImputEHR: A Visualization Tool of Imputation for the Prediction of Biomedical Data
Source: Front Genet. 2021 Jul 2;12:691274. doi: 10.3389/fgene.2021.691274 (PMC8283820; doi:10.3389/fgene.2021.691274)

Supplementary documents for “ImputeEHR: A visualization tool for imputation and prediction of biomedical data”

Dr. Yi-Hui Zhou

Supplementary Table 1. parameters for ImputeEHR and Missforest methods

Variables	Missforest	ImputeEHR1	ImputeEHR2	Title in Web app	Description
min_samples_split	Present			Minimum Sample Split	The minimum number of samples required to split an internal node.
min_samples_leaf	Present			Minimum Sample Leaf	The minimum number of data points allowed in a leaf node in the algorithm.
min_weight_fraction_leaf	Present			Minimum Weight Fraction Leaf	Similar to 'Minimum Sample Leaf', but it applies a minimum weighted fraction of the sum total number of observations instead.
max_iter	Present	Present	Present	Maximum Iterations	Maximum number of iterations of the algorithms for a single run.
n_estimators	Present	Present	Present	Number of Estimators	Number of trees in the forest.
min_impurity_decrease	Present			Minimum purity decreases	"A node will be split if this split induces a decrease of the impurity greater than or equal to this value". It controls deep of the tree growth based on the impurity.
min_child_weight		Present	Present	Minimum Child Weight	Minimum sum of instance weight needed in a child.
feature_fraction		Present		Feature Fraction	Randomly select a subset of features on each tree.
min_data_in_leaf		Present		Minimum Data in leaf	Minimal amount of data in one leaf.
learning_rate		Present	Present	Learning Rate	The weighting of new trees added to the mdoel based on previous training.
reg_alpha		Present	Present	Alpha	Penalizes the features which increase cost function.
reg_lambda		Present	Present	Lambda	Penalizes the features which increase cost function.
colsample_bytree			Present	Colsample by tree	"The subsample ratio of columns when constructing each tree."
max_depth			Present	Maximum Depth	Maximum depth of a tree.
subsample			Present	Subsample	The fraction of samples used to train each tree.
seed			Present	Random Seed	Generates a random number.

Supplementary Table 2. RMSE comparison on "Boston" dataset

Method	Missing rate								
	10%	20%	30%	40%	50%	60%	70%	80%	90%
ImputeEHR1	0.114978	0.125669	0.131543	0.145737	0.156552	0.178869	0.2038	0.240381	0.334493
ImputeEHR2	0.119488	0.127686	0.133542	0.149478	0.15845	0.17754	0.197838	0.225183	0.289165
MissForest	0.116216	0.126019	0.135121	0.14965	0.160782	0.188001	0.210132	0.236703	0.290227
KNNImputer	0.130386	0.141042	0.154849	0.18641	0.205988	0.220712	0.223168	0.227356	0.250472
Median	0.2552	0.258971	0.26155	0.262656	0.262049	0.25937	0.262208	0.262347	0.262792
Mean	0.239368	0.242788	0.244379	0.246505	0.245302	0.24435	0.245288	0.246513	0.247111
MICE	0.150927	0.163225	0.188969	0.190492	0.191048	0.199886	0.217161	0.238869	0.251581
GAIN	0.193608	0.202213	0.212916	0.217647	0.231566	0.253582	0.26303	0.277679	0.318002
SoftImpute	0.181574	0.195291	0.207958	0.222319	0.252619	0.297081	0.366145	0.432083	0.489495

Supplementary Table 3. RMSE comparison on "Spam" dataset

Method	Missing rate								
	10%	20%	30%	40%	50%	60%	70%	80%	90%
ImputeEHR1	0.043476	0.045346	0.047294	0.049896	0.052011	0.055027	0.058272	0.061724	0.06471
ImputeEHR2	0.045988	0.047445	0.049193	0.051399	0.053587	0.056377	0.059243	0.06278	0.065122
MissForest	0.04399	0.047092	0.050191	0.053819	0.057227	0.061816	0.065375	0.067067	0.069153
KNNImputer	0.050997	0.052679	0.055003	0.058369	0.061663	0.064872	0.065446	0.064661	0.063212
Median	0.059629	0.059329	0.058985	0.059096	0.059088	0.059385	0.059392	0.05945	0.059294
Mean	0.056999	0.056688	0.05628	0.05645	0.056493	0.056607	0.056683	0.05676	0.056714
MICE	0.050486	0.051262	0.056948	0.057137	0.056194	0.055832	0.056319	0.058356	0.058378
GAIN	0.053115	0.053143	0.052992	0.053393	0.05415	0.076926	0.117163	0.145616	0.104705
SoftImpute	0.052078	0.052219	0.052463	0.053321	0.05415	0.055495	0.057024	0.058801	0.060229

Supplementary Table 4. RMSE comparison on the "Letter" dataset

Method	Missing rate								
	10%	20%	30%	40%	50%	60%	70%	80%	90%
ImputeEHR1	0.060806	0.067738	0.07717	0.089248	0.104438	0.121849	0.140853	0.15895	0.172634
ImputeEHR2	0.076696	0.082775	0.090782	0.100749	0.113711	0.128302	0.144447	0.159544	0.170586
MissForest	0.055183	0.064853	0.076762	0.08979	0.105914	0.123275	0.142273	0.161177	0.175413
KNNImputer	0.052886	0.070103	0.103327	0.137558	0.151708	0.156251	0.158406	0.159188	0.158866
Median	0.156307	0.155951	0.15612	0.15601	0.156044	0.155986	0.15615	0.156443	0.156595
Mean	0.154239	0.153921	0.154105	0.153995	0.154104	0.154017	0.154159	0.154111	0.154166
MICE	0.107606	0.113959	0.129738	0.127899	0.129818	0.134936	0.145602	0.15143	0.152866
GAIN	0.125423	0.125861	0.129078	0.134452	0.141141	0.170261	0.21737	0.193009	0.198253
SoftImpute	0.125018	0.129601	0.135587	0.142208	0.151484	0.171541	0.227058	0.317721	0.401673

Supplementary Table 5. RMSE comparison on the "Breast cancer" dataset

Method	Missing rate								
	10%	20%	30%	40%	50%	60%	70%	80%	90%
ImputeEHR1	0.054102	0.059443	0.062588	0.06736	0.072117	0.079668	0.090277	0.108474	0.159389
ImputeEHR2	0.049758	0.057231	0.059362	0.065189	0.070451	0.078833	0.088787	0.106939	0.141309
MissForest	0.053336	0.060735	0.063845	0.069421	0.075033	0.084164	0.094558	0.113032	0.148652
KNNImputer	0.070186	0.076825	0.081365	0.088359	0.098691	0.11452	0.128722	0.133389	0.141395
Median	0.149228	0.1494	0.149541	0.148605	0.147469	0.147957	0.148466	0.148468	0.149669
Mean	0.144553	0.145027	0.144955	0.144406	0.143852	0.143922	0.144599	0.144539	0.14572
MICE	0.044473	0.054202	0.05792	0.065211	0.073206	0.08658	0.114337	0.132005	0.144713
GAIN	0.065866	0.071422	0.075858	0.081983	0.088645	0.102827	0.112339	0.142069	0.214848
SoftImpute	0.052617	0.058264	0.062131	0.068209	0.074203	0.084292	0.116503	0.18532	0.259543

Supplementary Table 6. Parameters of ImputeEHR methods

ImputeEHR1		ImputeEHR2	
**Parameter**	**Value**	**Parameter**	**Value**
feature_fraction	0.4	colsample_bytree	0.4
bagging_fraction	0.4	gamma	0
min_data_in_leaf	5	learning_rate	0.1
max_depth	-1	min_child_weight	1.5
learning_rate	0.1	eval_metric	rmse
boosting_type	gbdt	subsample	0.6
metric	rmse	max_depth	3
reg_alpha	0	reg_alpha	0
reg_lambda	0	reg_lambda	0
n_estimators	100	n_estimators	100

Supplementary Figure 1 The histogram of the missing rate of the toy example

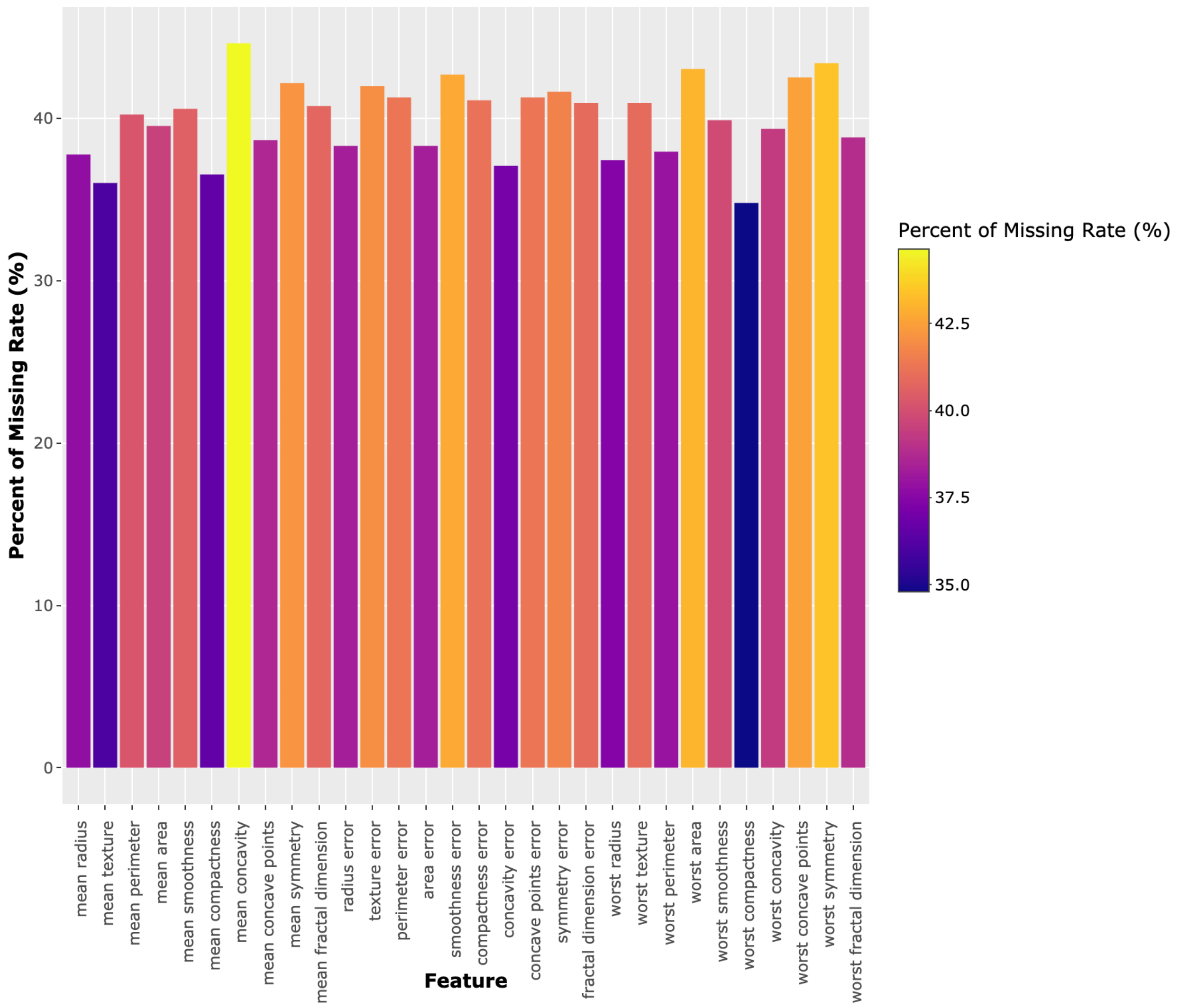

Percent of Missing Rate (%)
0
10
20
30
40
Feature
mean radius
mean texture
mean perimeter
mean area
mean smoothness
mean compactness
mean concavity
mean concave points
mean symmetry
mean fractal dimension
radius error
texture error
perimeter error
area error
smoothness error
compactness error
concavity error
concave points error
symmetry error
fractal dimension error
worst radius
worst texture
worst perimeter
worst area
worst smoothness
worst compactness
worst concavity
worst concave points
worst symmetry
worst fractal dimension
Percent of Missing Rate (%)
42.5
40.0
37.5
35.0

Supplementary Figure 2 The scatter plot between the pairwise features

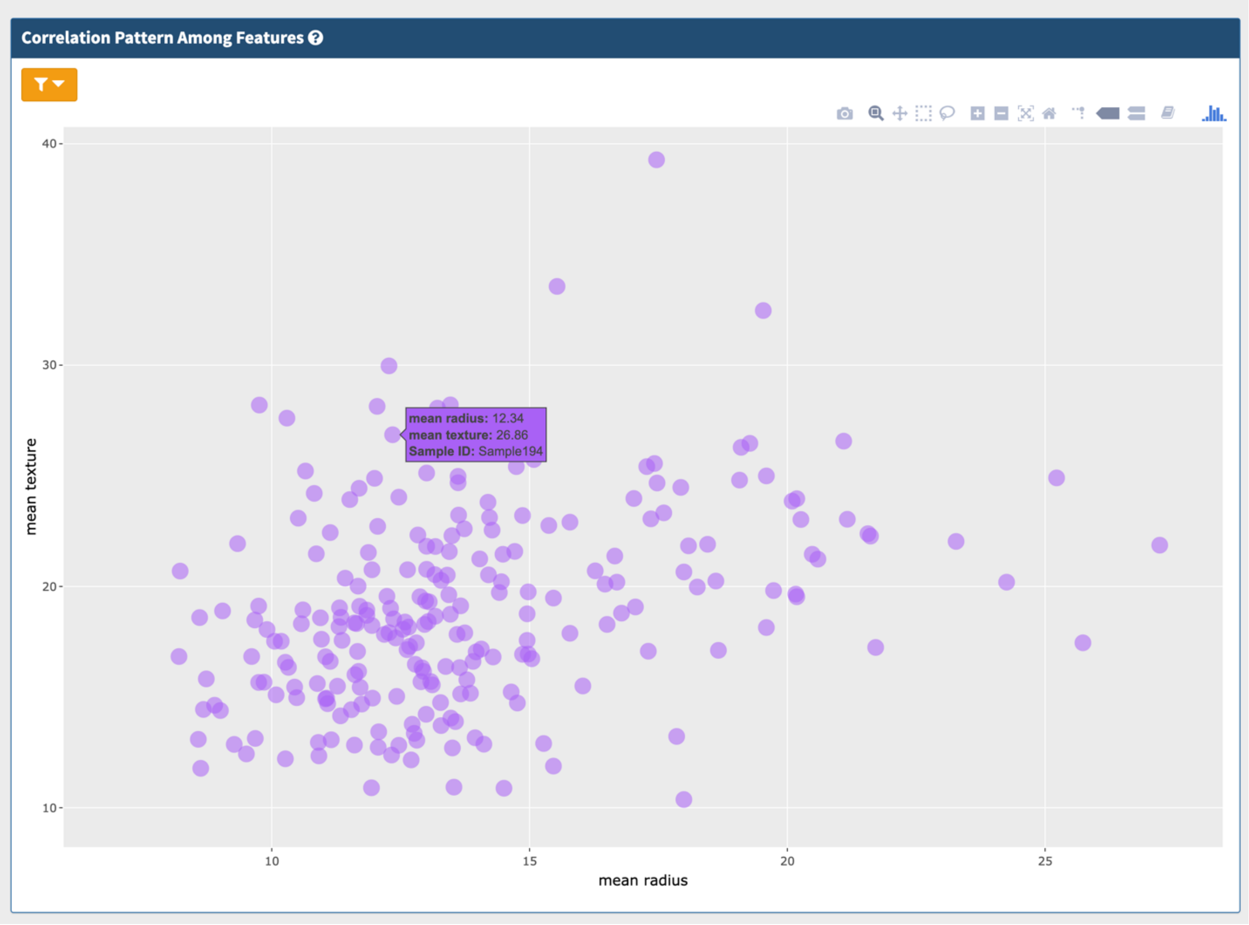

Supplementary Figure 3. Visualization of missingness patterns

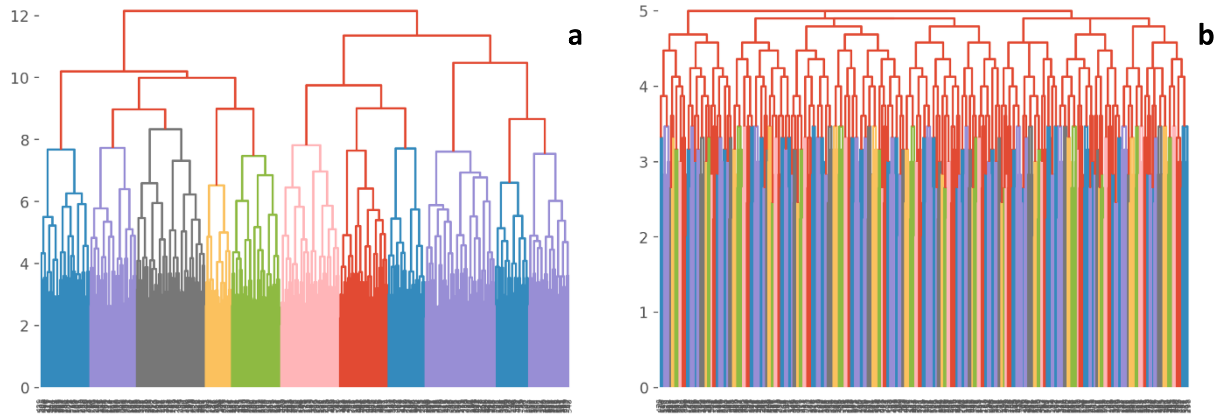

Visualization of Dendrogram on missingness pattern toy example dataset with two linkage methods: a) ward, and b) complete

Supplementary Figure 4: Elbow method for the best K in the section of visualization of missingness patterns

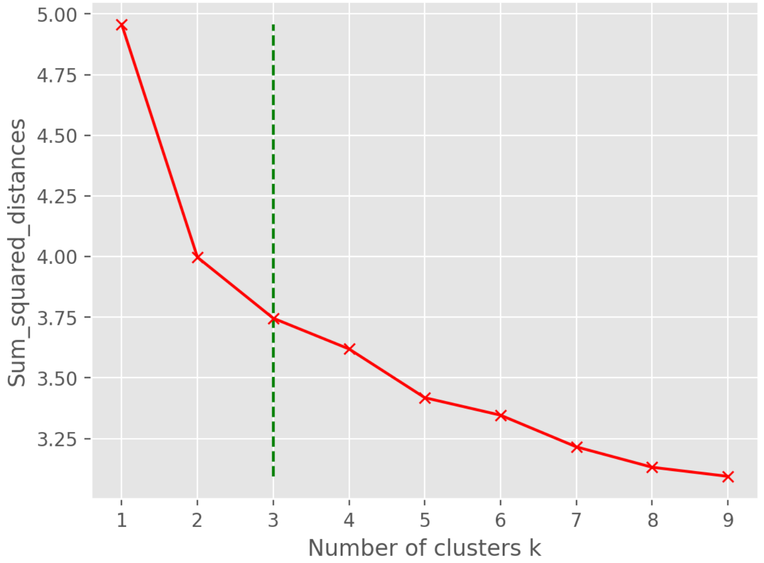

Supplementary Figure 5:
Combination of Dimensional Reduction and K-means Clustering

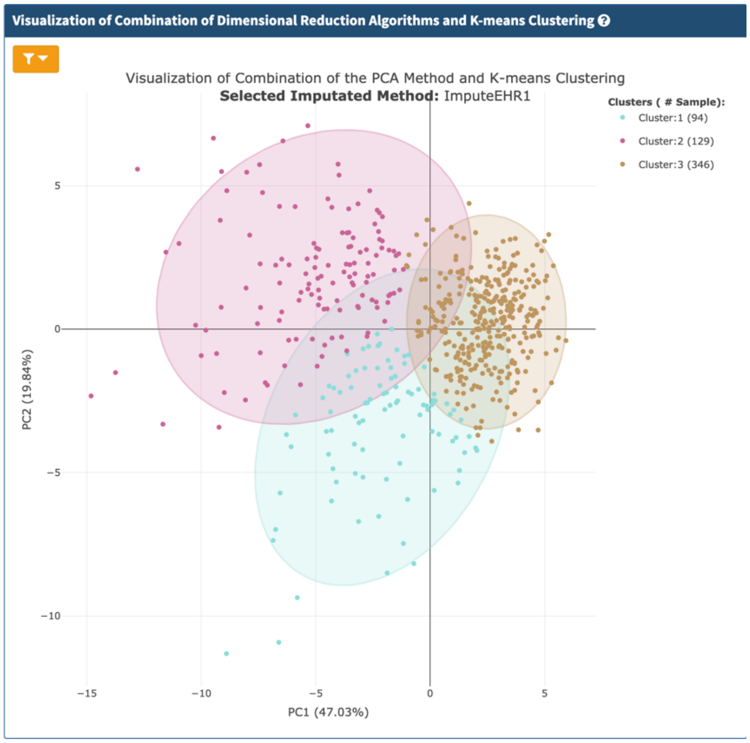

Supplementary Figure 6: the correlation between the imputed value and the masked 5% non-missing data, based on the toy example

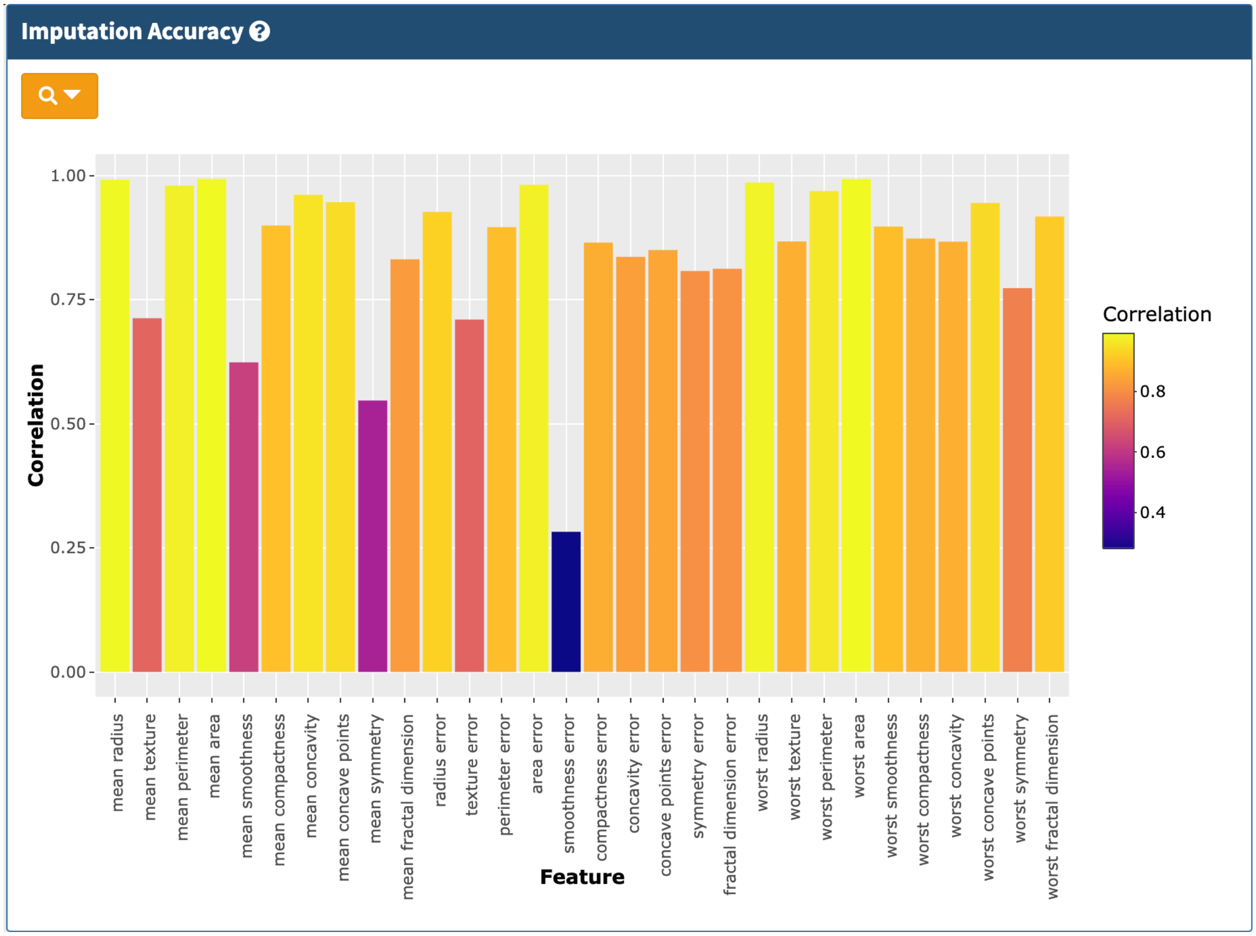

Supplement: Supplementary file 1 [file Data_Sheet_1.PDF]
